# Reachability Improvement of a Climbing Robot Based on Large Deformations Induced by Tri-Tube Soft Actuators

**DOI:** 10.1089/soro.2018.0115

**Published:** 2019-08-02

**Authors:** Ayato Kanada, Fabio Giardina, Toby Howison, Tomoaki Mashimo, Fumiya Iida

**Affiliations:** ^1^ Department of Engineering, Toyohashi University of Technology, Toyohashi, Japan.; ^2^ Department of Engineering, University of Cambridge, Cambridge, United Kingdom.

**Keywords:** continuum robots, soft actuators, climbing, nonlinear deformation modeling

## Abstract

Locomotion of soft-bodied organisms, such as amoeba, worms, and octopuses, is safe, robust, and adaptable and has great promise for applications in complex environments. While such organisms fully exploit the potential provided by their soft structures, engineering solutions commonly constrain soft deformation in favor of controllability. In this study, we study how soft deformations can enhance the climbing capabilities of a robot. We introduce a robot called Longitudinally Extensible Continuum-robot inspired by Hirudinea (LEeCH), which has few shape constraints. Inspired by real leeches, LEeCH has a flexible extensible body and two suction cups at the ends. It is capable of performing 3D climbing locomotion using two suction cups driven by vacuum pumps and tri-tube soft actuators which have only three DC motors. The large deformations occurring in LEeCH extend its workspace compared to robots based on constant curvature models, and we show successful locomotion transition from one surface to another at angles between 0° and 180° in experiment. We develop a model based on multibody dynamics to predict the nonlinear deformations of the robot, which we verify in the experiment. The model reveals a nondimensional morphological parameter, which relates the robot's shape to its mass, stiffness, and size. The workspace of LEeCH as a function of this parameter is studied in simulation and is shown to move beyond that of robots based on constant curvature models.

## Objective

Climbing robots have a wide range of potential applications, including building inspection, maintenance, construction, and search and rescue tasks.^1–4^ A challenging problem in climbing robots is increasing reachability to navigate and transition between obstacles such as steps and walls. Most climbing robots are yet to achieve such tasks, whereas soft-bodied animals such as leeches, slugs, and caterpillars easily complete them. One strategy often observed in such organisms is the exploitation of large deformations and, therefore, nonlinearities to increase reachability. Many climbing robots inspired by animals have been developed, but they mainly have focused on adhesion-based climbing inspired by, for example, gecko's van der Waals forces^5,6^ or insect spikes.^7–9^

Some robots that can climb at many angles and transition from wall to wall have been demonstrated. RAMR1,^10^ W-Climbot,^11^ and Shady3D^12^ (modular), which are biped robots that consist of several joints connected in series and two grippers attached to the ends, achieved transition from ground to wall and wall to ceiling. The high number of degrees of freedom can not only overcome obstacles and steps but also provide good dexterity when used as a manipulator. However, this comes at the cost of complicated control and larger torques. MultiTrack^13^ is composed of serially connected modules that have a caterpillar track with suction cups and can climb over a thin wall, that is, transitioning from wall to wall. It combines high mobility and maneuverability due to continuous locomotion and active joints. However, such a combination of multiple devices leads to complex controllability and a weight increase. Stickybot,^5^ Wallbot,^6^ and the Tank-Like robot^14^ are bioinspired robots using flat or fibrillar dry adhesives that can attach to various surfaces with low power consumption. Even though such robots successfully performed some transitions,^15^ there are some remaining issues such as loss of adhesion due to dirt. Since all climbing robots always have a risk of falling from large heights, being lightweight and flexible are desirable for safety and survival.

Unlike the aforementioned traditional robots with rigid links, soft robots have a great potential to interact with environments safely and adaptively.^16–19^ Some soft climbing robots with extreme compliance have been reported,^20–22^ but they can only generate simple locomotion on the wall. Despite the difficulties of modeling and control of soft robots arising from the many degrees of freedom in such systems, a few recent and very notable exceptions partially overcame these difficulties. Flippy^23^ is a cable-driven continuum robot with two grippers attached to the ends. It can transition between interior planes in different orientations by bending its body 180°. While this locomotion enables transition motion without complex sensing or control, its stride is restricted and increases the risk of collision with obstacles. Treebot^24^ has a continuum body that consists of three mechanical springs, and it can extend and bend in any direction by controlling the spring lengths. This provides a large working space and makes it possible to climb from a tree trunk to a branch. Treebot has superior maneuverability and adaptability, but the body deformation was only explored within the regime of deformations which was predicted by a constant curvature model, thus constraining the range of possible robot postures. It is still an open challenge to achieve a wall-to-wall transition in soft robots and to model and control large nonlinear deformations.

**FIG. 1.**
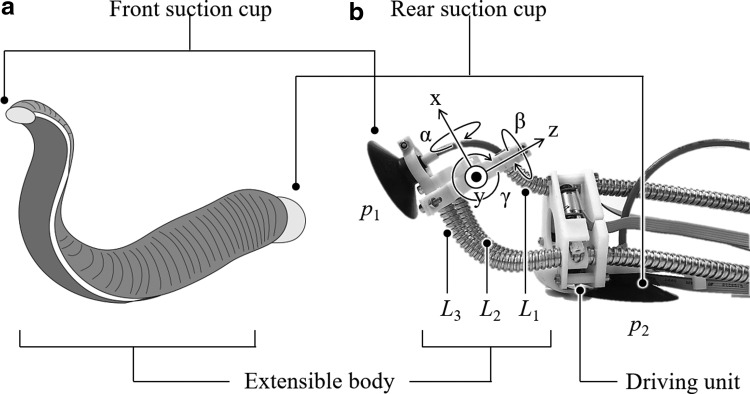
Schematic of proposed continuum robot. **(a)** Real leech. **(b)** LEeCH. LEeCH is a five DoF system, including two suction cups (*p*_1_ and *p*_2_), driven by pumps and three flexible tubes (*L*_1_, *L*_2_, and *L*_3_) controlled by DC motors. DoF, degree of freedom; LEeCH, Longitudinally Extensible Continuum-robot inspired by Hirudinea.

In this article, we describe a continuum robot design, which is inspired by leeches, belonging to the subclass Hirudinea, to address the challenges of achieving the wall-to-wall transition. Leeches are excellent climbers, propelling themselves using soft extensible bodies and two suckers attached at both ends. They can elongate their body greatly and traverse confined spaces. Our proposed bioinspired robot called LEeCH (Longitudinally Extensible Continuum-robot inspired by Hirudinea) adopts the leech's suckers and flexible and extensible body, as shown in Figure 1. With this morphology come the advantages and problems associated with the leech's motion control. While the flexibility allows for adaptive behavior, it also adds virtually infinite degrees of freedom, which need to be controlled for coordinated motion. The leech uses longitudinal, circular, and oblique muscles, which enable versatile body motions, including elongation and bending. We mimic this behavior with what we call a tri-tube soft actuator, consisting of three flexible tubes with helical grooves and a driving unit with three DC motors. Unsurprisingly, the full range of motion of the leech cannot be matched in our robot with only three degrees of actuation. For example, behaviors like twisting and body inflation are not directly achievable with the tri-tube soft actuator. However, the simple mechanism enables elongation/shortening and bending in all spatial dimensions, which we found to be sufficient for the rudimentary movements of leech-like climbing. LEeCH therefore integrates the skill of body extension, bending, and attachment to surfaces together with the flexibility of a leech's body.

The first step in exploiting the power of nonlinear deformations is to understand the physics behind deformation. In this work we present a model for large deformations to predict LEeCH's body shape. The constant curvature model is the most widely used kinematic framework,^25^ and a variety of ways for deriving the homogeneous transformation have been studied such as D–H parameters,^26,27^ Frenet–Serret formulas,^26^ and integral representation.^28^ However, it restricts the achievable workspace of the end effector. In addition, Bernoulli–Euler beam theory^29^ and Cosserat rod theory^30^ have been used to describe continuum robots whose shapes are controlled primarily by elasticity. We describe a model based on multibody mechanics,^31^ which involves masses, springs, and dampers in discrete links.

In general, improved flexibility contributes to robustness but restricts reachability due to nonlinear deformations such as buckling. However, we deem nonlinear deformations to be important for environment adaptation, which, for instance, can improve downward reachability under gravity. To verify our hypothesis, we validated this robot design and model through four experiments: (1) to investigate how accurately our model can predict the body shape of the real robot with large deformations; (2) to clarify in what cases a difference between our model and the constant curvature model appears; (3) to demonstrate six types of locomotion, including wall-to-wall transition; and (4) to compare the advantage in reachability with our model with the constant curvature model.

## Materials and Methods

### Model

**FIG. 2.**
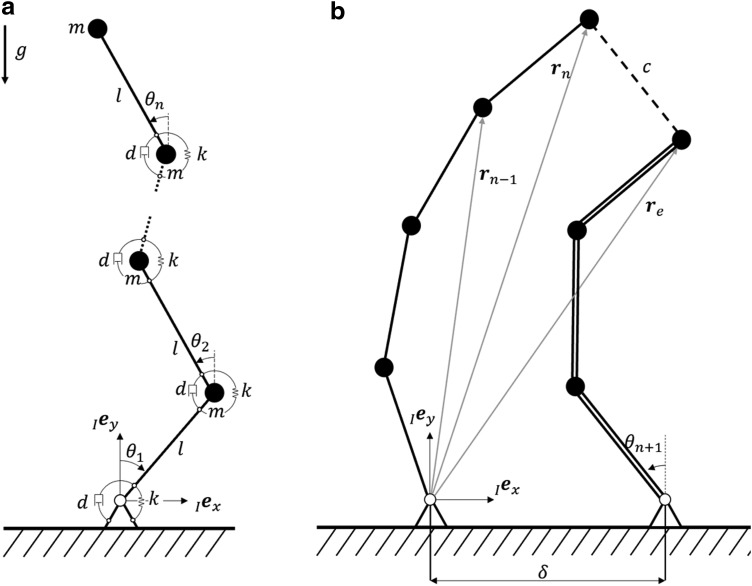
Sketches of the proposed model. **(a)** One chain of rigid bodies with generalized coordinates $\textbf{\textit{q}} = { \left[ {{ \theta _1} , { \theta _2} , \ldots , { \theta _n}} \right] ^T}$ and mass *m*, length *l*, stiffness *k*, and damping *d* per body. **(b)** Two chains located a distance $\delta$ apart and connected at their tips with geometric constraints.

The bending motions investigated in LEeCH are flat-wall climbing and wall-to-wall transitions. These motions occur only in one plane at a time, which allows for an accurate description of the system with a planar model. To approximate the soft body, we use a chain of rigid bodies under the influence of gravity with acceleration *g*. We assign a linear torsional stiffness *k* and linear damping *d* at every joint. The first element is attached with a joint to an inertial reference system *I*. Each rigid body is associated with a mass *m* located at its tip and a length *l*. Figure 2a illustrates such a chain of *n* elements.

The equations of motion are derived using the projected Newton–Euler equations with the generalized coordinates $\textbf{\textit{q}} = { \left[ {{ \theta _1} , { \theta _2} , \ldots , { \theta _n}} \right] ^T}$, that is, the sum of all *n* bodies' generalized momenta render the inertial terms of the equations of motion

\begin{align*}
\mathop \sum \limits_{k = 1}^n { \bf{J}}_k^T{ \dot
{\textbf{\textit{p}}_k}} = { \bf{M}} \ddot {\textbf{\textit{q}}} +
{\textbf{\textit{h}}}, \tag{1}
\end{align*}

where ${{ \bf{J}}_k}$ is the Jacobian of the point mass in body *k*, ${ \dot {\textbf{\textit{p}}_k}}$ is the time derivative of the momentum of the point mass in body *k*, ${ \bf{M}}$ is the mass matrix, $\ddot {\textbf{\textit{q}}}$ is the second derivative of the generalized coordinates, and ***h*** is the vector of gyroscopic accelerations.

#### Equations of motion

The equations of motion take the form

\begin{align*}
{ \bf{M}} \ddot {\textbf{\textit{q}}} + {\textbf{\textit{h}}} =
{\textbf{\textit{E}}} + {\textbf{\textit{G}}} + { \bf{J}}_c^T
\lambda , \tag{2}
\end{align*}

where ***E*** is the vector of external forces acting on the system, ***G*** is the vector of the generalized gravitational force, and ${ \bf{J}}_c^T \bm{\lambda}$ are the constraint forces. External forces can be applied at any point *i* on the model and are computed by

\begin{align*}
{\textbf{\textit{F}}_i} = {{ \bf{J}}_i}{\textbf{\textit{f}}_i} , \tag{3}
\end{align*}

with ${{ \bf{J}}_i}$ the Jacobian of the system at point *i* and *f_i_* the planar force applied at point *i*. Furthermore, external forces contain the stiffness and damping terms due to the springs and dampers we apply to the chain. The *i*th link in the chain is subject to the forces

\begin{align*}
{S_i} = - k \left( {{ \theta _i} - { \theta _{i - 1}}} \right) - k \left( {{ \theta _i} - { \theta _{i + 1}}} \right). \tag{4}
\end{align*}

and

\begin{align*}
{D_i} = - d \left( {{{ \dot \theta }_i} - {{ \dot \theta }_{i - 1}}} \right) - d \left( {{{ \dot \theta }_i} - {{ \dot \theta }_{i + 1}}} \right). \tag{5}
\end{align*}

Note that the first and the last element in the chain are described by

\begin{align*}
{S_1} = - k{ \theta _1} - k \left( {{ \theta _1} - { \theta _2}} \right). \tag{6}
\end{align*}

\begin{align*}
{S_n} = - k \left( {{ \theta _n} - { \theta _{n - 1}}} \right). \tag{7}
\end{align*}

and

\begin{align*}
{D_1} = - d{ \dot \theta _1} - d \left( {{{ \dot \theta }_1} - {{ \dot \theta }_2}} \right). \tag{8}
\end{align*}

\begin{align*}
{D_n} = - d \left( {{{ \dot \theta }_n} - {{ \dot \theta }_{n - 1}}} \right). \tag{9}
\end{align*}

The spring and damper generalized force terms can therefore be written as a matrix-vector multiplication by $${ \bf{S}}{\textbf{\textit{q}}} + { \bf{D}} \dot
{\textbf{\textit{q}}}$$ which leads to the external force vector

\begin{align*}
{\textbf{\textit{E}}} = {{ \bf{J}}_i}{\textbf{\textit{f}}_i} + {
\bf{S}}{\textbf{\textit{q}}} + { \bf{D}} \dot
{\textbf{\textit{q}}}. \tag{10}
\end{align*}

For the generalized gravitational force, we have

\begin{align*}
\textbf{\textit{G}} = \mathop \sum \limits_{i = 1}^n { \bf{J}}_i^T \left( { \begin{matrix} 0 \\ { - mg} \\ \end{matrix} } \right). \tag{11}
\end{align*}

As we will see in the next section “Robot model,” we model the robot with parallel chains of rigid bodies that are interacting through geometric constraints which are enforced on the equations of motion by Lagrange multipliers. Assume that we have defined a geometric constraint $\textbf{\textit{g}} \left( \textbf{\textit{q}} \right) = 0$. The second derivative of this constraint with respect to time takes the form

\begin{align*}
\ddot {\textbf{\textit{g}}} = {{ \bf{J}}_c} \ddot
{\textbf{\textit{q}}} + \bm{\xi} \left( {\textbf{\textit{q}} ,
\dot {\textbf{\textit{q}}}} \right) = 0. \tag{12}
\end{align*}

with ${{ \bf{J}}_c} = {{ \bf{J}}_c} \left( \textbf{\textit{q}} \right)$ what we call the constraint Jacobian and $\bm{\xi}$ containing all the terms not depending on $\ddot {\textbf{\textit{q}}}$. We will now make use of Gauss' principle to enforce the constraints in the equations of motion. First recall that we need to find the constraint force $\bm{\lambda}$ given in the equations of motion

\begin{align*}
\ddot {\textbf{\textit{q}}} = {{ \bf{M}}^{ - 1}} \left(
{\textbf{\textit{E}} + {\textbf{\textit{G}}} + { \bf{J}}_c^T
\bm{\lambda} - {\textbf{\textit{h}}}} \right). \tag{13}
\end{align*}

With (12) we obtain

\begin{align*}
{{ \bf{J}}_c}{{ \bf{M}}^{ - 1}} \left( {\textbf{\textit{E}} + \textbf{\textit{G}} + { \bf{J}}_c^T \bm{\lambda} - \textbf{\textit{h}}} \right) = - \bm{\xi}. \tag{14}
\end{align*}

Solving for $\lambda$ we get

\begin{align*}
\bm{\lambda} = { \left( {{{ \bf{J}}_c}{{ \bf{M}}^{ - 1}}{ \bf{J}}_c^T} \right) ^{ - 1}} \left( { - \bm{\xi} - {{ \bf{J}}_c}{{ \bf{M}}^{ - 1}} \left( {\textbf{\textit{E}} + \textbf{\textit{G}} - \textbf{\textit{h}}} \right) } \right). \tag{15}
\end{align*}

Inserting the found $\bm{\lambda}$ back to the equations of motion leads to the constrained equations of motion.

#### Robot model

For the chain of links to represent a model of our robot we add a second chain located a distance $\delta$ from the first one apart as shown in Figure 2b. Note that we doubled the stiffness, damping, and mass to model two tubes instead of just one in the second chain. This way, we can model the motion of our 3-tube robot in the plane. The interaction between the two chains is implemented by geometric constraints as outlined previously. More precisely, we added three constraints to model a bridge between the last elements of each link:

\begin{align*}
{\rm Parallel}: {\textbf{\textit{g}}_1} \left( \textbf{\textit{q}} \right) = { \theta _n} - { \theta _e}. \tag{16}
\end{align*}

\begin{align*}
{\rm Distance}: {\textbf{\textit{g}}_2} \left( \textbf{\textit{q}} \right) = {r_{ne}} - c. \tag{17}
\end{align*}

\begin{align*}
{\rm Perpendicular}: {\textbf{\textit{g}}_3} \left(\textbf{\textit{q}} \right) = \textbf{\textit{r}}_{n \left( {n - 1} \right) }^T{\textbf{\textit{r}}_{ne}}. \tag{18}
\end{align*}

The parallel constraint ensures that the last two elements of each chain, element *n* on the first chain and element *e* on the second, are parallel to each other. The distance constraint enforces the tip of each chain to be a distance *c* apart from each other. Finally, the perpendicular constraint makes sure that the last link of the first chain is perpendicular to the distance vector of the two chain tips.

The robot's suction cup with mass *m_s_* is affecting its behavior and we thus model it as an external force acting on the element *e* on the second chain with

\begin{align*}
{\textbf{\textit{F}}_{{m_s}}} = { \bf{J}}_e^T \left( { \begin{matrix} 0 \\ { - {m_s}g} \\ \end{matrix} } \right). \tag{19}
\end{align*}

**FIG. 3.**
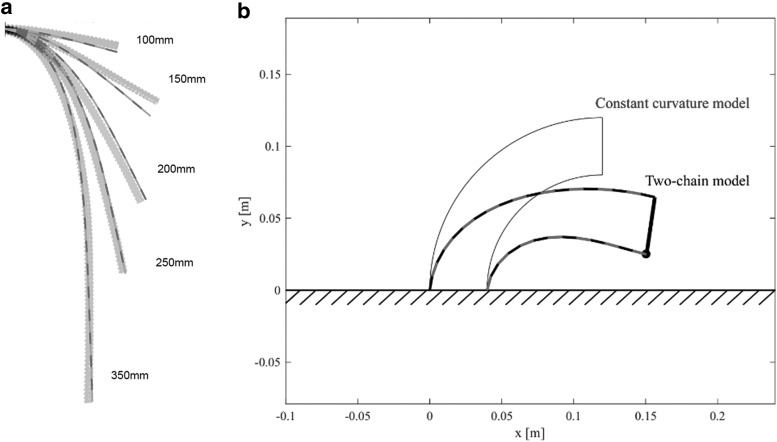
Simulation results of the model. **(a)** Comparison of the static solution of the model in simulation with the real-world tube for different tube lengths. **(b)** Comparison of the two-chain model with the constant curvature model.

For the subsequent investigation we have used model parameters as indicated in Table 1. We found the stiffness by comparing the model prediction of a chain with a tube experiment, where we measured the slacking of the tube under the influence of gravity for different tube lengths, as shown in Figure 3a. The element mass is simply found by weighing the tube and dividing it by the element length.

**Table 1.** Values of Physical Parameters

*Parameter*	*Symbol*	*Value*
Stiffness	*k*	$0.055 { \frac { { \rm { Nm } } } { { \rm { rad } } } }$
Element mass	*m*	$0.0068 \;{ \rm{kg}}$
End effector mass	*m_s_*	0.021 kg
Chain distance bottom	$\delta$	$0.0398 \;{ \rm{m}}$
Chain distance top	*c*	$0.0398 \;{ \rm{m}}$
Element length	*l*	$0.01 \;{ \rm{m}}$
Gravitational acceleration	*g*	$9.81 { \frac { \rm { m } } { { { \rm { s } } ^2 } } }$

The presented model is dynamic, but since we are interested in the static solution, we will compute the forward dynamics in simulation until we reach the static equilibrium. Therefore, we do not need to get an accurate value for the damping terms from the real system, but need to make sure that the simulation converges to the static solution. In most of our simulations we therefore chose a value of $0.1 { \frac { { \rm { Nms } } }  { { \rm { rad } } } }$ which was underdamped but converged to the equilibrium in a reasonable time (shown in Supplementary Video S1).

Figure 3b shows the simulation result of the proposed model with a constant curvature model. The chains in both models have the same length, but the proposed model is bending under the effect of gravity due to its stiffness, constraints, and inertial properties.

### Design and control

#### Robot design

**FIG. 4.**
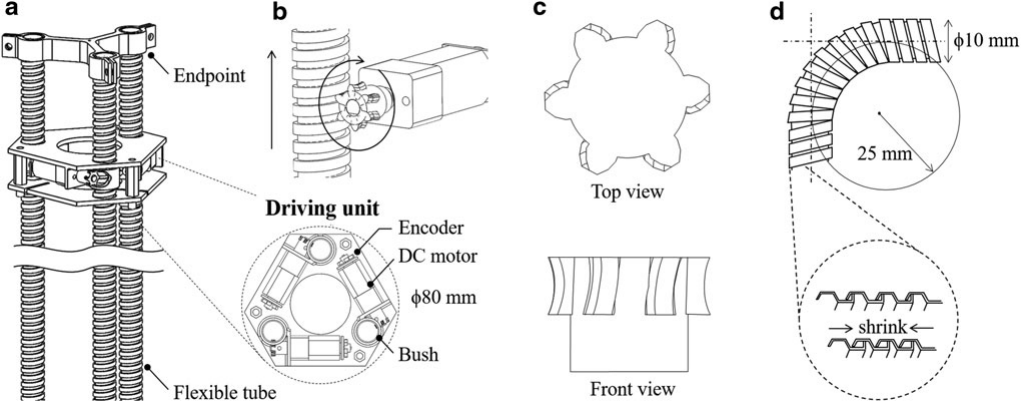
Tri-tube soft actuator diagram. **(a)** Overview. **(b)** Rack and pinion mechanism. **(c)** Enveloping worm gear. **(d)** Flexible tube.

Figure 4a shows an overview of tri-tube soft actuators, which are a proposed continuum body composed of three flexible tubes that are connected in parallel.^32^ The flexible tubes, which are located at the vertices of an equilateral triangle, pass through a driving unit with three DC motors (75:1 Micro Metal Gearmotor HP 6V, Pololu Co.). One end of tube is fixed to an endpoint made of plastic, and the other end is free. A gear attached to the DC motor engages with the helical groove on the surface of the flexible tube. As with the rack and pinion mechanism, the rotational motion moves the flexible tube laterally relative to the driving unit, as shown in Figure 4b. The continuum body can bend or elongate by controlling the length of each flexible tube. To improve the engagement between the gear and flexible tube, the gear was designed to be similar to an enveloping worm, whose diameter increases from its center toward the end and whose teeth twist clockwise along the axis, as shown in Figure 4c. The flexible tube, called stripwound metal hose, has been used to protect electrical wires or liquid and gas tubes. The flexible design is formed by spirally winding a metal plate with S-shaped profile, as shown in Figure 4d. The bending motion is achieved because each S-shaped corrugation slides against each other and contracts. We adopted a flexible tube with a diameter of 10 mm and a minimum bending radius of 25 mm.

Although we can use a helical spring instead of the flexible tube as shown in Refs.,^33,34^ the engagement with the gear would not be robust because of the elasticity. For example, when a continuum robot climbing a wall supports its weight by a mechanism attached to the endpoint, the tubes or springs are subject to a heavy load. Consequently, the flexible tube does not elongate, whereas the spring elongates.

#### Robot architecture and locomotion principle

**FIG. 5.**
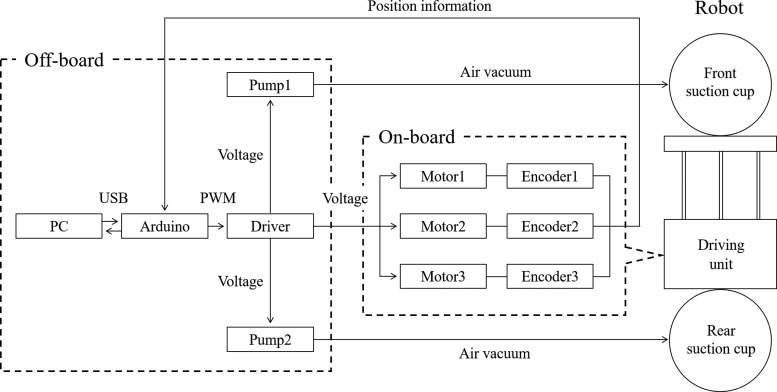
System hierarchy.

Figure 5 shows the control system of the proposed robot. A personal computer hosting the user interface is connected to an Arduino Mega by USB and sends commands for specific motions. The Arduino generates a PWM signal based on the command and supplies voltages to motors and pumps through drivers. The front and rear suction cups with a diameter of 50 mm (ZP50CN; SMC Co., Japan) are actuated using two vacuum pumps (D2028B; AIRPON, China). In this suction system, the payload capacity per one suction cup is up to ∼1.7 kg on a vertical wall, and it is possible to lift our robot with a weight of 240 g (on-board). Each DC motor moves the flexible tube according to the applied voltage and elongates or bends the robot body. Encoders attached to the end of the motors send position information of the flexible tube to the Arduino, closing a feedback loop. Control in the present system does not need to be very precise and we therefore neglect additional effects arising in our soft-tube actuators, such as backlash, for the sake of simplicity. For tasks requiring high accuracy, however, the controller needs to consider such effects.

**FIG. 6.**
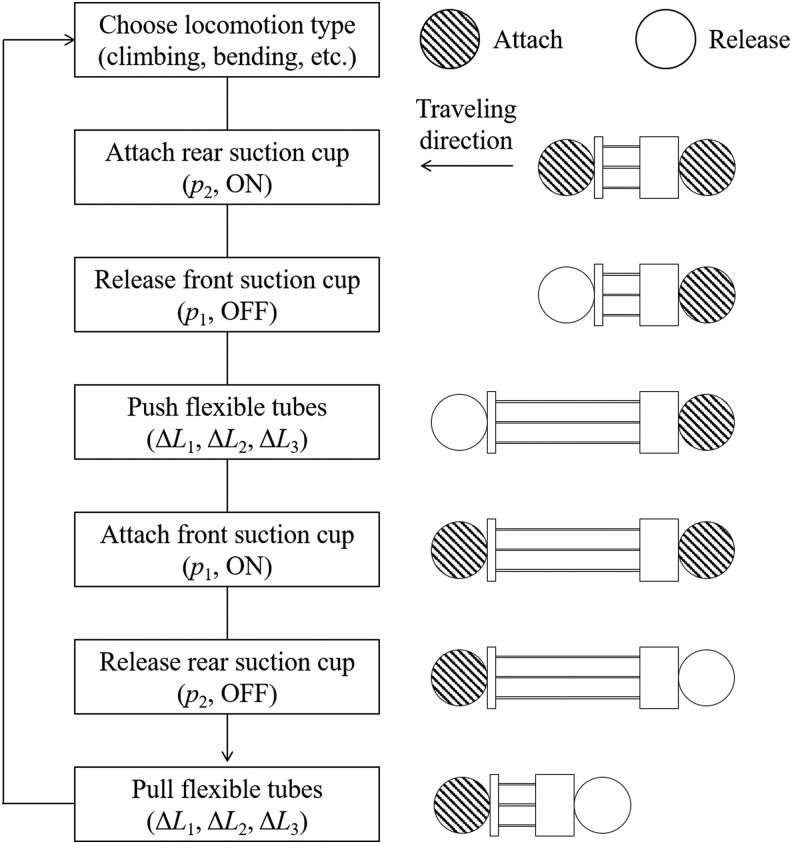
Steps in the locomotion procedure.

The locomotion of the proposed robot is similar to that of leeches and inchworms, which involves repeated elongation/contraction of the continuum body and releasing/grasping of the front and rear suction cups. The steps in the locomotion procedure are shown in Figure 6 and can be described as follows. The circle with hatch pattern represents the suction cup that attaches to the substrate, while the other white circle represents the suction cup that detaches to the substrate. First, we choose the locomotion type such as climbing and bending and set the flexible tube length (*L*_1_, *L*_2_, and *L*_3_). For example, when the robot moves while turning to the right, the length of the left tube *L*_2_ is set larger compared with the right tube *L*_3_. After the rear suction cup adheres to the substrate, the front suction cup releases. Then, each tube is pushed until it has the set length. At this time, if the length of the center tube *L*_1_ is short, the robot lifts its end, and if the tube on either side is short, the robot body turns to either left or right. After the front suction cup attaches to the substrate, the rear suction cup releases. The robot contracts by pulling all flexible tubes which completes one stride of the locomotion procedure.

## Results

### Model comparison

**FIG. 7.**
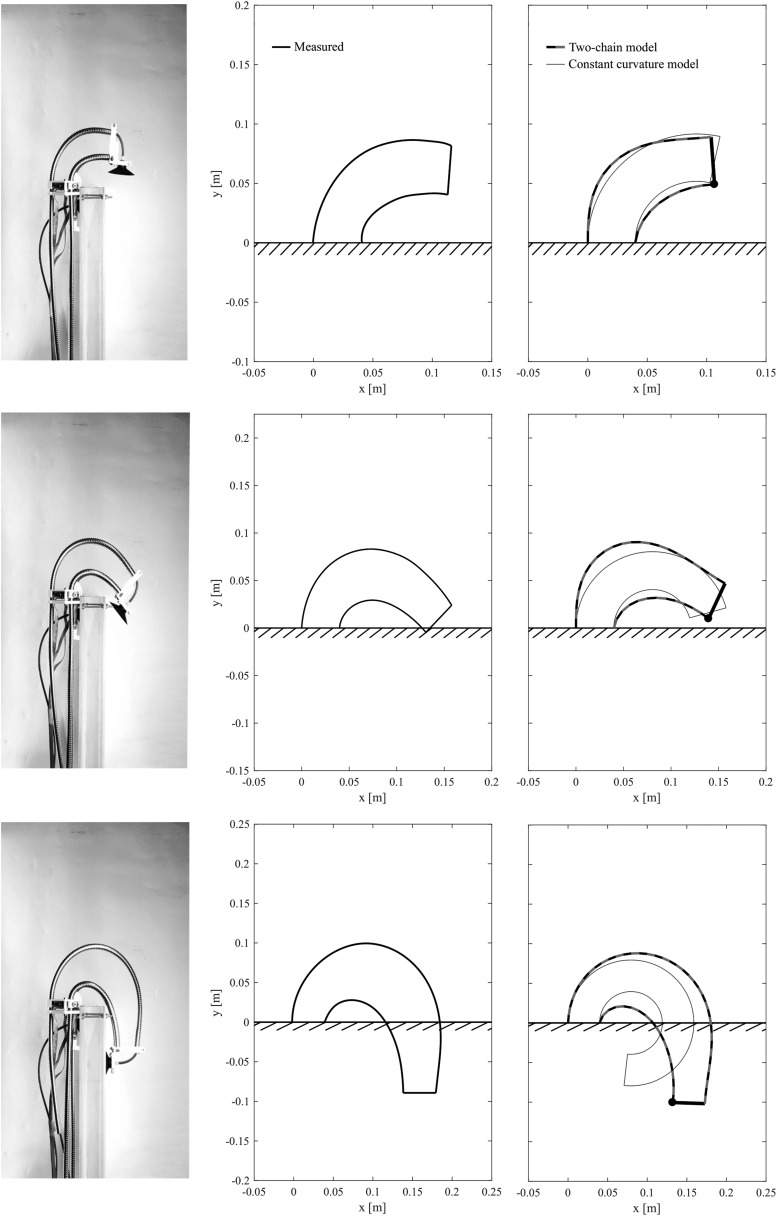
Comparison of the flexible tube shape in simulation with the real world. The *left images* show the real robots attached on the vertical wall. The *middle column* of figures shows a simplified representation of the real robot deformations, and the figures on the *right-hand side* show the two-chain model and the constant curvature model.

To evaluate the accuracy of the proposed model, we measured the deformations of the flexible tubes in our robot. A comparison of the robot shape in simulation with the real world is shown in Figure 7. In this experiment, we took several pictures while the robot was attached to a vertical wall. The tube shape was measured from the images and resized for comparison with the model. The stiffness of the model was tuned manually until the deformations of the model and the experiment were matched. Furthermore, we measured tube lengths from the picture images and substituted these values for the two models: The two-chain model and constant curvature model. When the tube lengths are short, there is almost no difference between these two models, whereas when the tube lengths are long, the tube shape and robot endpoint differ greatly due to the effect of gravity and internal elasticity. The proposed model predictions match the real robot deformations well, even when the tubes are greatly deformed. Minor errors between the real robot and the proposed model are detectable, which probably arise because of stiffness nonlinearities in the real system and neglected mass and hardness of the air supply tubes inside the central flexible tube.

### Nondimensional shape parameter

The constant curvature model is a kinematic model and, thus, describes the position of the end effector as a geometric property. In LEeCH, the position also depends on the internal stiffness, mass distribution, gravity, and length of the extendable robot segments. When looking at the moment balance of a single chain in our model, one can see that the element angles depend only on these four parameters. For an element *i*, the moment balance reads

\begin{align*}
mg \mathop \sum \limits_{j = i}^n {x_j} = - k \left( {{ \phi _i} - { \phi _{i - 1}}} \right) , \tag{20}
\end{align*}

where ${ \phi _0} = 0$. This is equal to

**FIG. 8.**
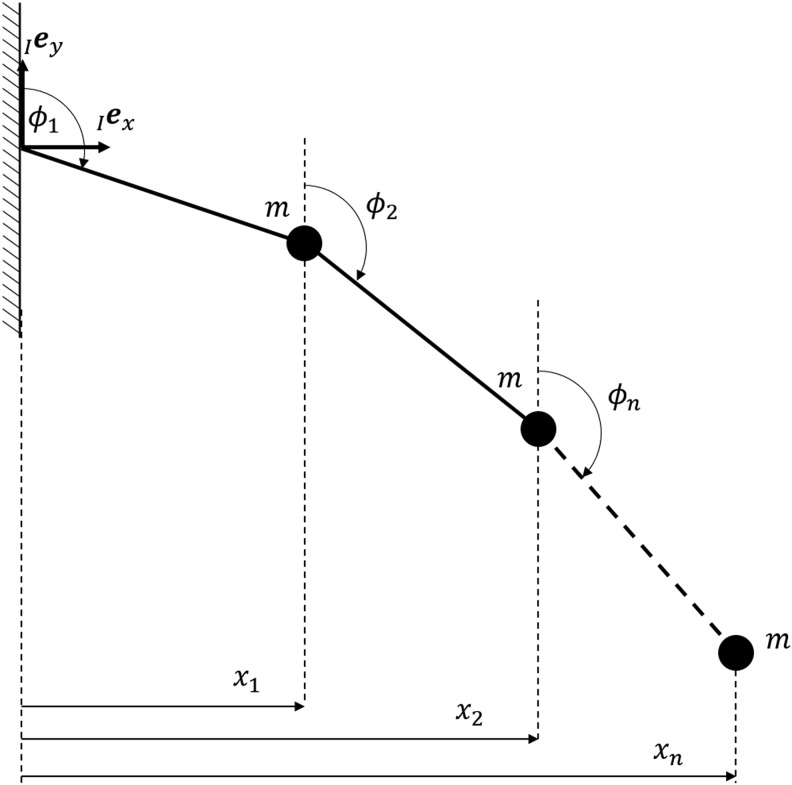
One-chain model of *n* elements used for the derivation of the shape parameter $\sigma.$

\begin{align*}
mg \mathop \sum \limits_{j = i}^n \mathop \sum \limits_{p = i}^j l \sin { \phi _p} = - k \left( {{ \phi _i} - { \phi _{i - 1}}} \right) , \tag{21}
\end{align*}

with *m* the mass per element, *g* gravity, *x_j_* the *x* coordinate of the mass *j* as shown in Figure 8, *l* the element length, *k* the torsional stiffness per element, and ${ \phi _i}$ the angle of element *i* with respect to the frame of reference *I*. Thus, we have for the angle of element *i*

\begin{align*}
- \frac { 1 }  { { \left( { { \phi _i } - { \phi _ { i - 1 } } } \right) } } \mathop \sum \limits_ { j = i } ^n \mathop \sum \limits_ { p = i } ^j \sin { \phi _p } = \frac { k }  { { mgl } } . \tag { 22 } 
\end{align*}

**FIG. 9.**
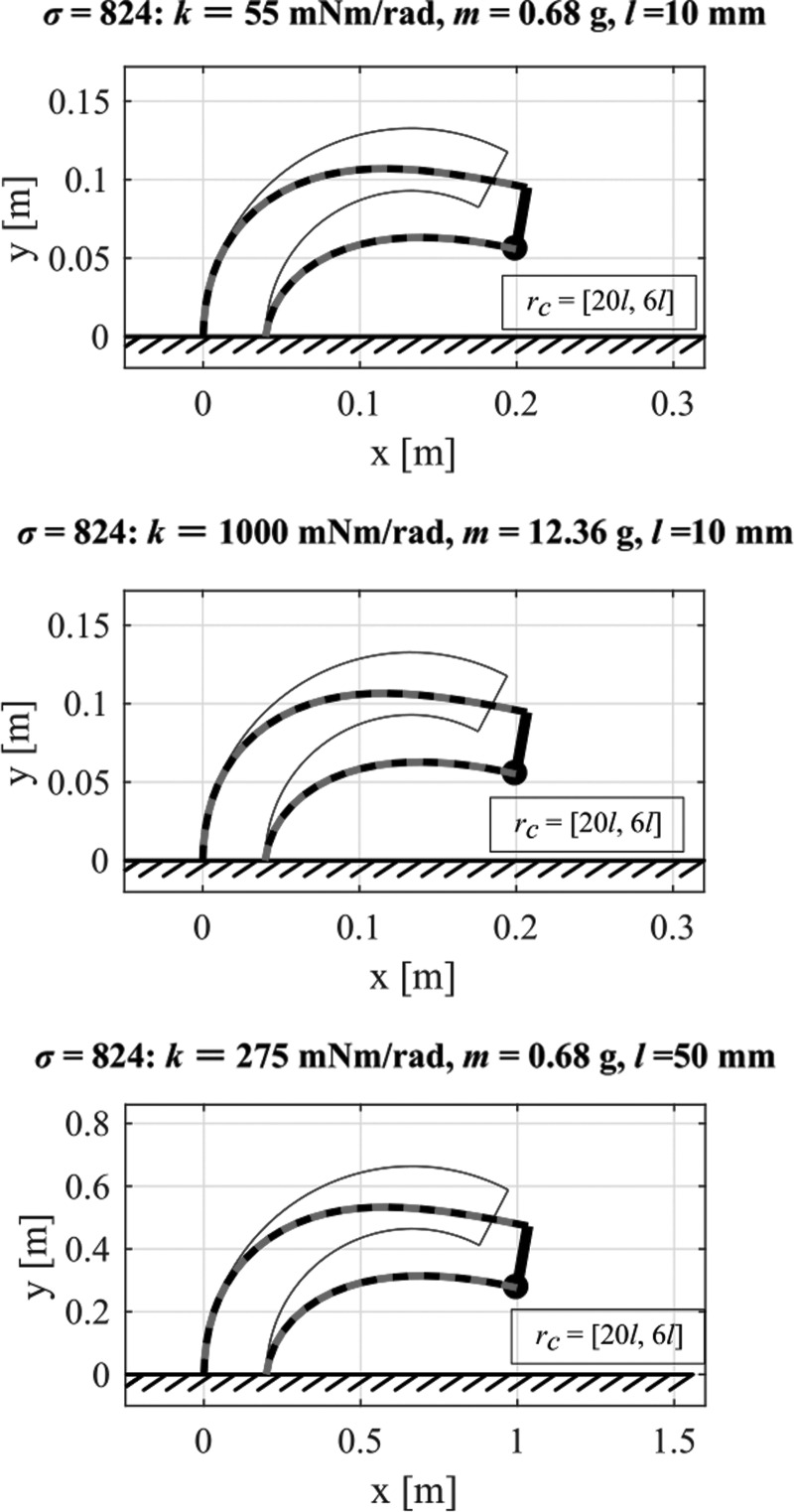
Deformation for constant shape parameter $\sigma$ but changing stiffness *k*, mass *m*, and element length *l*. The constant $\sigma$ guarantees the same robot shape under certain conditions such as the number of elements *n* does not change.

We see that the resulting angles are completely defined by the nondimensional parameter $k / mgl$ which we will refer to as the shape parameter $\sigma$ from here on. For any $\sigma$ which remains constant, the resulting angles stay constant under the conditions that the number of elements does not change, that the initial conditions are identical, and that the payload *m_s_* at the tip is zero. This means that we can, for instance, change the size of the robot by changing *l* and guarantee the same robot shape as long as we change the other parameters to keep $\sigma$ constant. Figure 9 illustrates this using our model and changing stiffness, mass, and element length but keeping $\sigma$ constant. We see that for all configurations the overall shape does not change although the size of the robot may. For all cases, the vector pointing to *r_c_* which is located at the tip of chain 2 is located at the coordinates [$20l \; , \;6l$] irrespective of size. This is particularly interesting in the third case where we changed the element length which led to a further reach than in the other cases. Note, however, that we also adapted the chain distance at the bottom and top by the same factor as the element length. Interestingly, such a scaling is relevant in the natural world. For example, although caterpillars increase their body weight by 10,000 times in 2 weeks, they maintain the same locomotion kinematics by changing muscle activation and therefore the stiffness *k.*^25^

**FIG. 10.**
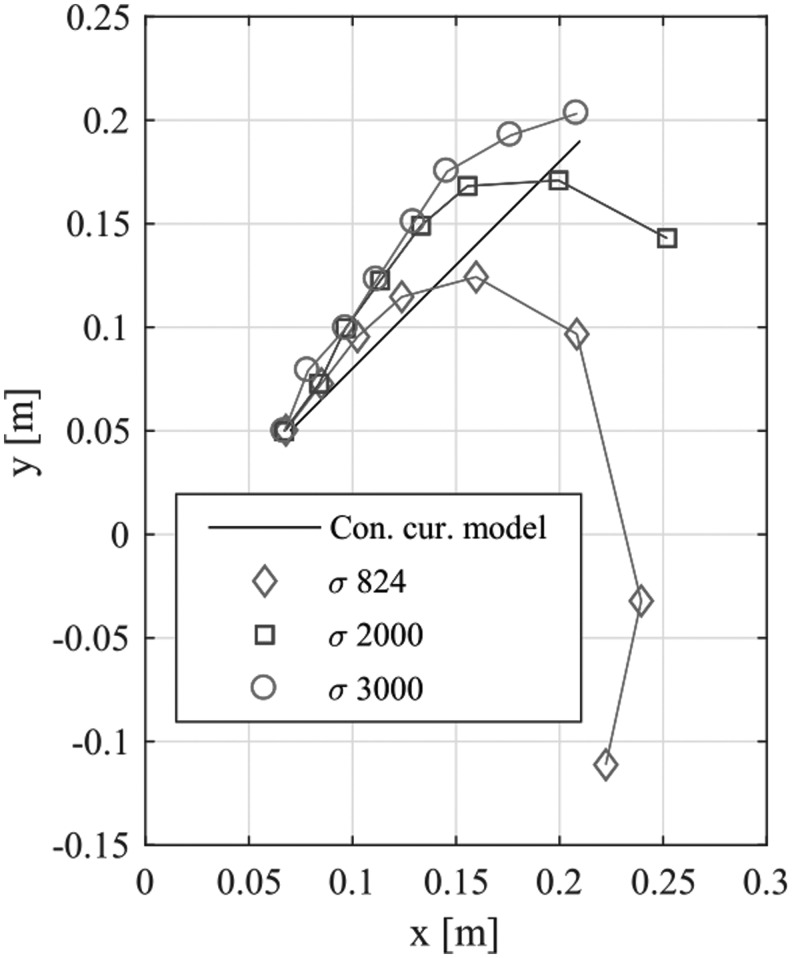
Comparison between the predicted endpoints of the constant curvature model and the two-chain model when changing the shape parameter $\sigma$ by changing stiffness *k* and mass *m*. The constant $\sigma$ guarantees the same stiffness of the robot as long as element length *l* and gravitational acceleration *g* are constant even when changing the robot size by changing the number of elements *n*.

Figure 10 shows how the two-chain model approaches the constant curvature model when stiffness and mass are changed, giving rise to different shape parameters $\sigma$. We set the endpoints of the constant curvature model to draw a linear curve and compared to that of our model with different stiffness parameters. The predicted endpoints in the simulation decline greatly due to the effects of gravity when $\sigma$ is low, that is, close to that of our real robot. As $\sigma$ increases, our model becomes stiffer and approaches the constant curvature model, but it does not match it completely. This is probably because the difference in the number of elements *n* and tubes in the left and right generates different bending moments in the two chains.

### Locomotion test

**FIG. 11.**
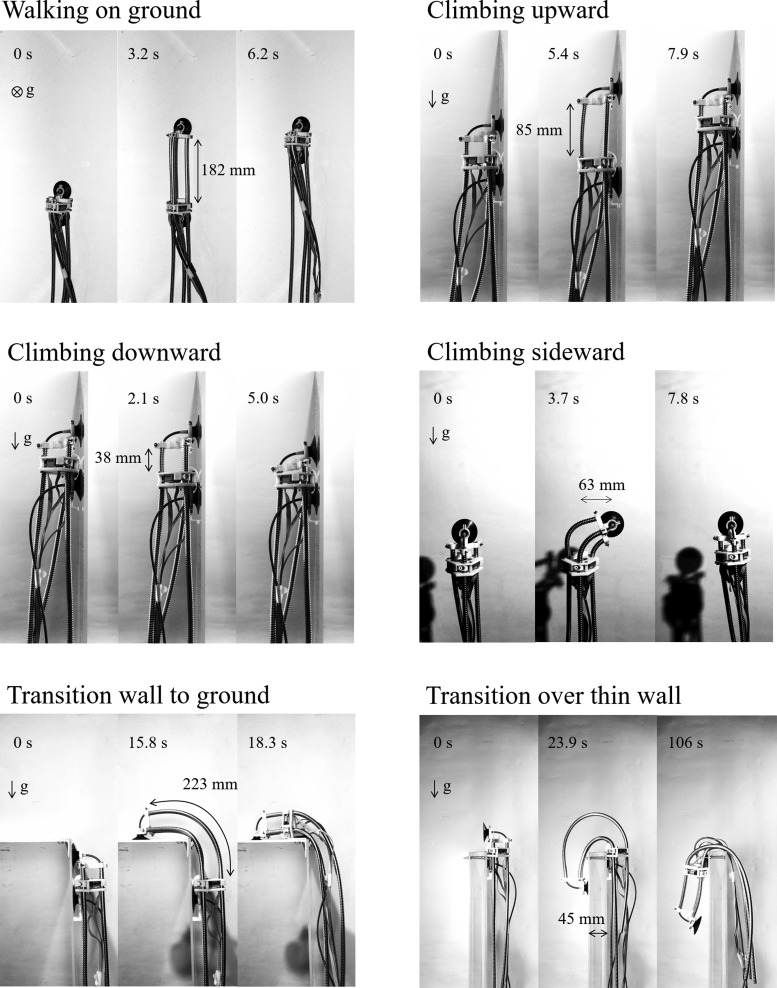
Six types of locomotion maneuvers.

The lengthening based on the rack and pinion mechanism using flexible tubes enables the robot to deform greatly, augmenting reachability in locomotion. To understand the locomotion behavior in our robot, six types of locomotion maneuvers were tested on a plastic plate as shown in Figure 11. On the flat ground, the tube length can be increased without buckling because the robot does not apply its load on the flexible tubes. A maximum speed of 20 mm/s was observed at a maximal tube length of 180 mm (left top figure and Supplementary Video S2).

The robot also achieved upward and downward climbing locomotion on a vertical wall (right top and left middle figures and Supplementary Videos S3 and S4). We can see that the tube lengths when climbing downward are shorter than when climbing upward. This is to guarantee that the rear suction cup is parallel to the wall at the point when it is being pressed against it. The middle right figure and Supplementary Video S5 show a sideway climbing maneuver. After the front suction cup with a free rotation joint is attached to the wall, the robot releases the rear suction cup and turns due to gravity, recovering the original posture. Note that this strategy for sideway climbing allows lateral movement but prevents steering of the entire robot (e.g., from vertical position to horizontal position) on vertical walls. If the free rotation joint is fixed the robot can achieve these movements, but the passive self-righting of the body posture upon release of the rear suction cup would be lost. The current version of our robot therefore possesses a rotational joint for simplified climbing control.

Locomotion for the transition from the vertical wall to the ground (90°–180° transition) and the climb over a wall (90°–270° transition) are described (bottom figures and Supplementary Videos S6 and S7). A climbing strategy which was not tested in our experiments is moving along a ceiling. This is currently not possible in our robot but can be achieved in future versions by improving the suction cup performance and reducing the robot weight. The robot was operated manually because when the tube lengths are long, autonomous control of the robot end is difficult due to its nonlinear deformation. An operator controls the motors to move the robot's end and looks for a touchdown position of the front suction cup. Note that the touchdown position of the front suction cup depends on that of the rear suction cup. The wall-to-ground transition and the thin wall transition take ∼18 and 86 s, respectively. The latter is relatively slow because the driving unit is caught on the corner when getting over the wall.

### Reachability

**FIG. 12.**
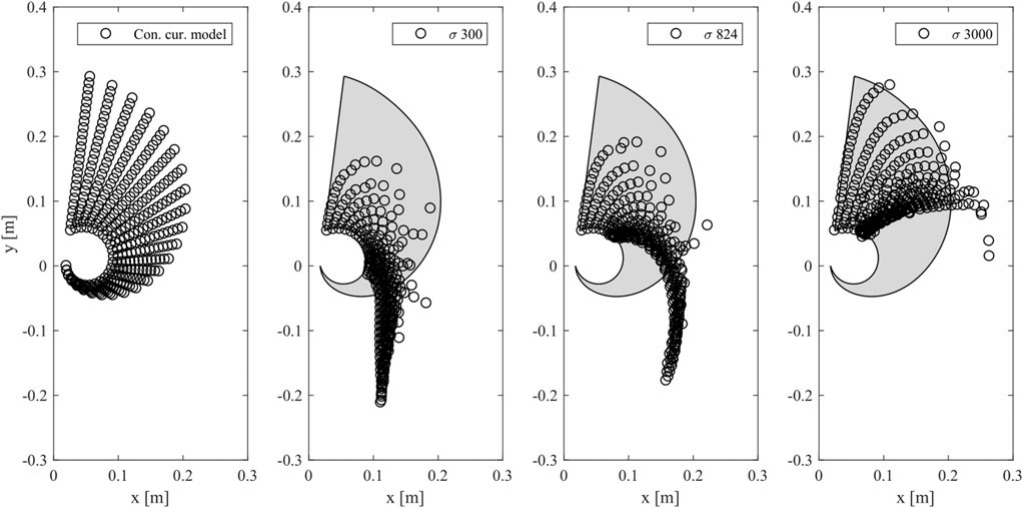
Manipulator workspace for constant curvature and two-chain model when changing the shape parameter $\sigma$ by changing only stiffness *k* and mass *m* as with Figure 10. *Circles* show the reachable points of the end effector, and the *gray area* indicates the reachable area of the constant curvature model.

In this study, we investigate to what extent the reachability with limited tube lengths is improved when using a multibody mechanics model as opposed to the constant curvature model. To do this, we set the range of each tube length from 0.05 to 0.3 m, varied each within this range, and numerically visualized the endpoints for each of these models for different shape parameter values $\sigma$ as shown in Figure 12. The shape parameter values are changed by changing only the element torsional stiffness *k* and element mass *m* as with Figure 10. Note that since we assume a scenario where the real robot gets over walls, the left tube is always longer than the right tube and the payload at the tip is set as *m_s_*.

We can see that the downward reachability is greatly increased using the two-chain model when $\sigma = 824$ or less, as it allows us to utilize the effect of gravity and tube bending. Note that $\sigma = 824$ corresponds to LEeCH's shape parameter when using the parameters in Table 1. Moreover, we can increase the upward reachability by increasing the shape parameter $\sigma$. This shows that we can design the robot's reachability by changing its shape parameter. Conversely, we see that the upward reachability is better in the constant curvature model, because gravity is not influencing the kinematic posture. It can also reach points in the neighborhood somewhat lower than the origin that cannot be accessed with the two-chain model.

The results show that both models have their unique advantages. While the constant curvature model can target specific points in space precisely, it does not take into account the effect of external forces and thus cannot exploit properties of the physical environment such as gravity. In the two-chain model, we can exploit the environment to extend its workspace, but cannot turn beyond 180° without help (e.g., environmental constraints). In continuum robots, both properties are needed depending on the problem. For example, accurate positioning is required in medical application such as endoscopes,^4^ while soft deformations are beneficial for moving and climbing in unstructured environments.^23,24^ In practical applications, stiffness parameters should be tunable depending on desired reachability. Even robots having poor upward reachability, that is low stiffness, can climb walls with the help of environmental constraints, but decreasing stiffness leads to difficult controllability of their body ends.

## Conclusion

To demonstrate the potential of a climbing robot with large deformation, we built a Longitudinally Extensible Continuum-robot inspired by Hirudinea, or LEeCH. An extensible body based on a rack and pinion mechanism can increase reachability of the robot end effector. The use of flexible tubes (stripwound metal hoses) as the rack showed great advantages for continuum robots: (1) flexible yet strong and (2) robust engagement with gears. Six types of locomotion, including wall-to-wall transition, were tested to demonstrate the robot's capability, and we found that large deformation is beneficial in certain situations. While our first prototype is inspired by the leech's basic morphology and locomotion, we are currently planning to adopt other features such as their special suckers and multiple muscles for grasping in unstructured substrates and achieving more complex tasks.^35^ Compared to the constant curvature model, the two-chain model based on multibody mechanics can accurately predict the real robot's deformation and improve the downward reachability under gravity. The motion studied in our work can be expressed in a planar model; however, more complex movements will involve rotations and motions out of the plane which require more degrees of freedom to be considered. In future work, we will extend our 2D model to 3D and allow for additional physical effects such as twist or stretch.

Moreover, we derived the nondimensional morphological parameter which defines the robot's shape and showed that the desired reachability can be designed by changing this parameter. In a real system, a change of shape parameter can be achieved by modifying system size, stiffness, mass density, or gravity. As the latter two are hard to alter and the system size might be constrained by the particular task at hand, a change in stiffness appears to be a reasonable tuning parameter, which could be achieved using variable stiffness actuators. In future work, we will investigate such mechanisms for live changes in shape parameters. Our study helps lay the foundation for soft robots that achieve complex locomotion such as overcoming obstacles and transitioning from wall to wall while using large and nonlinear deformation. A summary of our research is shown in Supplementary Video S8.

There are many other opportunities for future developments. For example, one problem is the reduction in controllability as the tube lengths increase, which makes it difficult to position the robot end. One way to improve controllability is to increase the number of the driving units controlling the flexible tubes. This allows the robot not only to assume various shapes but also to control the stiffness by changing the distance between the driving units. Such additional degrees of freedom will require an extension of our model to 3D, as out-of-plane bending, twist, and body inflation can occur which cannot be covered with our current planar model. Another opportunity for improvement is to prevent an unactuated helical degree of freedom of the flexible tubes. Even when a position of the driving unit is fixed, the axial rotation of the flexible tube is still possible due to the helical pitch of the tube grooves. Using a rotation stopper such as a set of a nut and slider used in lead screws may eliminate the rotational instability. Although this prototype robot has pumps, batteries, and a controller off-board, we could develop a self-contained robot by integrating these devices into on-board because increasing the weight of the driving unit does not affect the reachability of the robot end.

## Supplementary Material

Supplemental data

Supplemental data

Supplemental data

Supplemental data

Supplemental data

Supplemental data

Supplemental data

Supplemental data
